# Inflation and deflation pressure-volume loops in anesthetized pinnipeds confirms compliant chest and lungs

**DOI:** 10.3389/fphys.2014.00433

**Published:** 2014-11-10

**Authors:** Andreas Fahlman, Stephen H. Loring, Shawn P. Johnson, Martin Haulena, Andrew W. Trites, Vanessa A. Fravel, William G. Van Bonn

**Affiliations:** ^1^Life Sciences, Texas A&M University-Corpus ChristiCorpus Christi, TX, USA; ^2^Department of Anesthesia, Critical Care and Pain Medicine, Beth Israel Deaconess Medical CenterBoston, MA, USA; ^3^The Marine Mammal CenterSausalito, CA, USA; ^4^Vancouver AquariumVancouver, BC, Canada; ^5^Marine Mammal Research Unit, Fisheries Centre, University of British ColumbiaVancouver, BC, Canada; ^6^A. Watson Armour III Center for Animal Health and Welfare, Shedd AquariumChicago, IL, USA

**Keywords:** lung mechanics, total lung capacity, minimum air volume, excised lung, diving physiology

## Abstract

We examined structural properties of the marine mammal respiratory system, and tested Scholander's hypothesis that the chest is highly compliant by measuring the mechanical properties of the respiratory system in five species of pinniped under anesthesia (Pacific harbor seal, *Phoca vitulina;* northern elephant seal, *Mirounga angustirostris*; northern fur seal *Callorhinus ursinus*; California sea lion, *Zalophus californianus*; and Steller sea lion, *Eumetopias jubatus*). We found that the chest wall compliance (C_CW_) of all five species was greater than lung compliance (airways and alveoli, C_L_) as predicted by Scholander, which suggests that the chest provides little protection against alveolar collapse or lung squeeze. We also found that specific respiratory compliance was significantly greater in wild animals than in animals raised in an aquatic facility. While differences in ages between the two groups may affect this incidental finding, it is also possible that lung conditioning in free-living animals may increase pulmonary compliance and reduce the risk of lung squeeze during diving. Overall, our data indicate that compliance of excised pinniped lungs provide a good estimate of total respiratory compliance.

## Introduction

In 1940, Per Scholander described the unusual properties of the marine mammal respiratory system, and suggested that the highly compliant lung and rib cage would easily compress and shunt air into the rigid upper airway (Scholander, 1940). Having a compliant chest wall that empties to very low volumes (i.e., low functional residual capacity) would reduce the chances of extreme negative intra-thoracic pressures (lung squeeze), which are known to protect against pulmonary edema, cardiac arrhythmias and caval rupture (Leith, 1989).

Scholander's hypothesis is a central tenet in marine mammal diving physiology. An implicit assumption of his hypothesis is that the structural properties (compliance) of the respiratory system reduce uptake of N_2_ and the risk of decompression sickness (DCS). However, only a few studies have attempted to determine how pressure affects gas exchange in forced diving or freely diving marine mammals (Ridgway and Howard, 1979; Kooyman and Sinnett, 1982; Falke et al., 1985; McDonald and Ponganis, 2012, 2013). These studies revealed considerable differences among species in the estimated depth at which alveolar collapse occurred, but used different methods to quantify alveolar compression. Differences ranged from directly measuring the depth related pulmonary shunt (Kooyman and Sinnett, 1982), to indirectly measuring O_2_ or N_2_ tension in arterial and venous blood during a dive (Falke et al., 1985; McDonald and Ponganis, 2012, 2013), or measuring N_2_ removal from the muscle following a series of repeated dives (Ridgway and Howard, 1979).

A mathematical model was developed and calibrated against empirical data to understand alveolar collapse and how pressure affects pulmonary shunt in marine mammals (Bostrom et al., 2008; Fahlman et al., 2009). The objective of the model was to create a theoretical framework that could predict the air volumes of the various compartments of the respiratory system to the limit of collapse. Results suggested that the diving lung volume, the relative size, and the structural properties (compliance) of the upper and lower airways were important in determining the depth at which the alveoli collapse and gas exchange ceases (Bostrom et al., 2008). Comparing model output to available data for collapse depth in different species (Fahlman et al., 2007, 2009) led to the conclusion that behavioral (diving lung volume) and structural (lung and dead space compliance) variations between species could account for the observed species differences in estimated alveolar collapse depth.

Static pressure-volume (P-V) loops are commonly used to measure the physical properties (compliance) of the respiratory system. Published data exist on excised lungs for several terrestrial species, but only a few measurements have been made for marine mammals (Kooyman and Sinnett, 1979; Piscitelli et al., 2010; Fahlman et al., 2011). It is therefore difficult to assess the predictions of air volumes from the theoretical model without species-specific respiratory compliance estimates. In light of these challenges, we measured respiratory compliance in anesthetized pinnipeds to improve understanding of the structural properties of the marine mammal respiratory system, and to test Scholander's hypothesis that the chest is highly compliant.

## Materials and methods

### Animals

Static respiratory pressure and volume (P-V) data were collected opportunistically during scheduled veterinary procedures in four wild species of pinniped that were temporarily admitted to the Marine Mammal Center (TMMC, Sausalito, CA, harbor seal, *Phoca vitulina*; northern elephant seal, *Mirounga angustirostris*; northern fur seal, *Callorhinus ursinus*; and California sea lion, *Zalophus californianus*). Comparable data were obtained for one species raised in an aquatic facility under human care (Steller sea lion, *Eumetopias jubatus*, Table 1) during scheduled medical or diagnostic procedures unrelated to our research. All procedures on live animals were performed under inhalational anesthesia at the Marine Mammal Center (TMMC, Sausalito, CA), or the University of British Columbia and Vancouver Aquarium's Open Water Research Station (OWRS, Vancouver, Canada). Some animals had grave prognoses, e.g., cancer, broken jaw, and were euthanized. In those animals, the static P-V relationship was determined in the intact dead animal and/or of the excised lungs (Table 1). Some animals were anesthetized more than once, allowing the opportunity for repeated measurements. All work was approved by the IACUC at each institution (TAMUCC-IACUC AUP # 04-11; VanAqua 2012; TMMC # 2001–02).

**Table 1:** **Animal identification number (Animal ID), facility (The Marine Mammal Center, TMMC; Open Water Research Station, OWRS), group (Ph=phocid, Ot=otariid), sex (F-female, M-male), species (Pv = *Phoca vitulina*, Ma = *Mirounga angustirostris*; Cu = *Callorhinus ursinus*; Zc = *Zalophus californianus*; Ej = *Eumetopias jubatus*), body mass (*M*_b_), animal length, and compliance (L • cmH_2_O) for live (C_live_), intact dead animals (C_dead_) and excised lungs (C_exc_)**.

**Animal ID**	**Facility**	**Group**	**Sex**	**Species**	***M*b (kg)**	**Length (cm)**	**C_live_**	**C_dead_**		**C_exc_**	**TLC_est_**
							**(L • cmH_2_O)**				**(L)**
F97SI	OWRS	Ot	F	Ej	228	228	0.362		–	–	19.9
F97HA	OWRS	Ot	F	Ej	172	200	0.168		–	–	15.4
F00BO	OWRS	Ot	F	Ej	160	209	0.417		–	–	14.4
F00YA	OWRS	Ot	F	Ej	206	232	0.220		–	–	18.2
F03WI	OWRS	Ot	F	Ej	171	221	0.432		–	–	15.3
F03RO	OWRS	Ot	F	Ej	161	211	0.107		–	–	14.5
					**183 ± 28**	**217 ± 12**	**0.284 ± 0.138**				
CSL10244	TMMC	Ot	M	Zc	126	166	0.924^*^		–	–	11.6
CSL10301	TMMC	Ot	F	Zc	12	84	–		–	0.056	1.3
CSL10320	TMMC	Ot	M	Zc	18	106	–		0.205	–	1.9
CSL10325	TMMC	Ot	M	Zc	107	116	–		–	0.258	9.9
CSL10328	TMMC	Ot	M	Zc	75	146	–		–	0.197	7.2
CSL10638	TMMC	Ot	M	Zc	49	133	0.154		0.171	–	4.8
CSL10650	TMMC	Ot	M	Zc	17	121	0.128			–	1.8
CSL10653	TMMC	Ot	M	Zc	39	140	0.295		0.247	–	3.9
					**55 ± 43**	**126 ± 25**	**0.375 ± 0.373**		**0.208 ± 0.038**	**0.170 ± 0.104**	
NFS266	TMMC	Ot	F	Cu	53	125	0.575		–	–	5.2
HS2250	TMMC	Ph	F	Pv	8	45	–		–	0.021	0.9
HS2258	TMMC	Ph	M	Pv	10	83	0.112		–	–	1.1
HS2266	TMMC	Ph	F	Pv	10	81	0.116		–	0.087	1.1
					**9 ± 1**	**70 ± 21**	**0.114 ± 0.003**			**0.054 ± 0.047**	
ES3354	TMMC	Ph	F	Ma	50	124	0.300		–	–	4.9
ES3418	TMMC	Ph	F	Ma	42	129	0.140^*^		–	–	4.2
					**46 ± 6**	**126 ± 4**	**0.220 ± 0.113**				

* *P_tp_ < 10 cmH_2_O. Compliance estimates for excised lungs are from deflation data. Bold values under each species is mean (± 1 SD) value*.

### Morphometrics

Each animal was weighed (± 0.2 kg) and routine morphometric measurements were completed before the start of each procedure (Table 1). Measurements included standard length (cm, all animals) and axillary girth (seals only) and only length is reported (Table 1).

### Static and dynamic pressure-volume measurements of live pinnipeds

All wild animals were sedated using an intra-muscular injection of midazolam and butorphanol or midazolam and alfaxolone and manually restrained before anesthetic induction using isoflurane (1–4% isoflurane and O_2_), while the Steller sea lions were trained to enter a squeeze cage and fitted with a face-mask through which anesthetic gas (1–5% isoflurane) was delivered (Table 1). All animals were intubated using a human (Hudson RCI, Teleflex Medical Inc.) or veterinary (Equine Nasotracheal Tube, Jorgensen Laboratories) endotracheal tube of suitable size; and either breathed voluntarily or were ventilated while sedated and kept anesthetized throughout the procedure using isoflurane in O_2_.

Ventilatory flow-rates were measured using a pneumotachometer (3813 series, 0–800 l/min, Hans-Rudolph Inc.) placed in-line with the endotracheal tube. The pneumotachometer was connected to a differential pressure transducer (MPX-2.5 mbar type 339/2, Harvard apparatus, Holliston, MA) and calibrated for flow-rate using a 3 or 7 L calibration syringe (Series 5530 or 4900, Hans-Rudolph Inc, Shawnee, KS). A sample line attached to the endotracheal tube measured airway pressure (P_aw_) using a differential pressure transducer (MPX-100 mbar type 339/2, Harvard apparatus, Harvard Apparatus, Holliston, MA). Esophageal pressure (P_es_) was measured using a balloon catheter (Cooper Surgical, Trumbull, CT) connected to a second differential pressure transducer (MPX-100 mbar type 339/2, Harvard apparatus, Holliston, MA). The catheter was inserted into the esophagus so that the balloon was placed at the level of the heart, and inflated with 1 ml of air. Ambient pressure (P_amb_) was used as a reference for P_aw_ and P_eso_ and set at 0 cmH_2_O. The differential pressure transducers were connected to an amplifier (Tam-A, Harvard apparatus, Holliston, MA), and the data captured at 200 Hz using a Powerlab data acquisition system (8/35, ADInstruments, Colorado Springs, CO) and displayed on a laptop using LabChart (v. 7.3.7, ADInstruments, Colorado Springs, CO).

Animals either breathed spontaneously or were manually ventilated using an anesthesia bag or mechanically ventilated using an anesthesia ventilator (Hallowell EMC Model 2002 Anaesthesia Ventilator) and all variables measured from the tracheal intubation tube. For the current study only data from manual ventilations were used to estimate the static respiratory compliance (lung and chest). During manual ventilation, the lungs were inflated with a known volume (VT) of gas (O_2_ or air) and together with the change in the transpulmonary pressure (ΔP_tp_ =ΔP_aw_—ΔP_eso_) the static lung compliance (C_L_ = VT • ΔP^−1^_tp_, L • cmH_2_O^−1^) was estimated.

### Static pressure-volume measurements post mortem

For animals that were scheduled for euthanasia, the endotracheal tube, esophageal catheter, and pneumotachometer were left in place and additional measurements were made immediately after death. The calibration syringe was connected in place of the ventilator and the lungs were then inflated by serial injections of air.

### Static pressure-volume measurements of excised lungs

The entire respiratory tract was excised (lungs, bronchi and trachea), with the heart attached. The excised tissues were placed on a tray and the endotracheal tube attached to a manifold of 3-way valves with the 3 L or 7 L volumetric calibration syringe, producing inflation of the lungs in increments of 100 ml. The P_aw_ was measured using the system as described above.

The lungs were initially pre-conditioned by at least 3 inflations to a transpulmonary pressure (P_tp_) of ~ 30 cmH_2_O (P_tp_ = P_aw_—P_amb_), which in mammals usually is defined as the volume at total lung capacity (TLC, Loring et al., 2007; Fahlman et al., 2011). The total volume used to inflate the lung to a P_tp_ of 30 cmH_2_O was divided in 4–5 equal increments. The P-V relationship was determined by adding or removing air in the increments determined during the pre-conditioning step. A minimum of 3 leak-free inflation/deflation curves were recorded. In most cases, we performed one last inflation/deflation cycle to a P_tp_ between 40 and 50 cmH_2_O. As the risk of over distending the alveoli increases at pressures > 30 cmH_2_O, the last inflation was not included in the analysis. After injection or removal of a bolus of air, the volume was held constant until the pressure had reached a plateau, usually for between 15 and 20 sec, before the next step-wise change in lung volume (TLC, Loring et al., 2007; Fahlman et al., 2011).

In a previous study (Fahlman et al., 2011), the average Minimum Air Volume (MAV), in excised marine mammal lungs was estimated at 7% of TLC. MAV is an estimate of the residual volume (RV), and also of Functional Residual Capacity (FRC), in animals in whom the chest does not resist compression, i.e., has very high compliance. We added MAV to all lung volumes to estimate the total air in the lung. Thus, if the chest had low compliance this may slightly underestimate the true volume.

### Data processing and statistical analysis

Room temperature and ambient pressure were used to convert all volumes to standard temperature pressure dry (STPD, BTPS = STPD × 0.862). We assumed intrapulmonary air to be saturated. The estimated total lung capacity (TLC_est_) was computed as Fahlman et al. (2011):

(1) $${\text{TLC}}_{\text{est}}=0.135•{\text{Mb}}^{0.92}$$

The relationship between pressure and volume (lung compliance) was estimated from the slope of the P-V curve at 50% TLC_est_. For excised lungs, we used the data for the deflation curve. As C_L_ varies with size (Kooyman, 1973; Fahlman et al., 2011), the specific lung compliance (sC_L_, cmH_2_O^−1^) was computed by dividing C_L_ by the MAV previously estimated in excised lungs (Stahl, 1967).

Analysis of Variance (ANOVA) was used to compare differences in compliance with body mass and between groups. In this study, *P*-values ≤ 0.05 were considered as significant, whereas *P*-values ≤ 0.1 were considered to be suggestive of a possible trend. Data are presented as mean ± standard error of the mean (SEM), unless otherwise stated.

## Results

### Static P-V relationship in live animals

**Figure 1:**
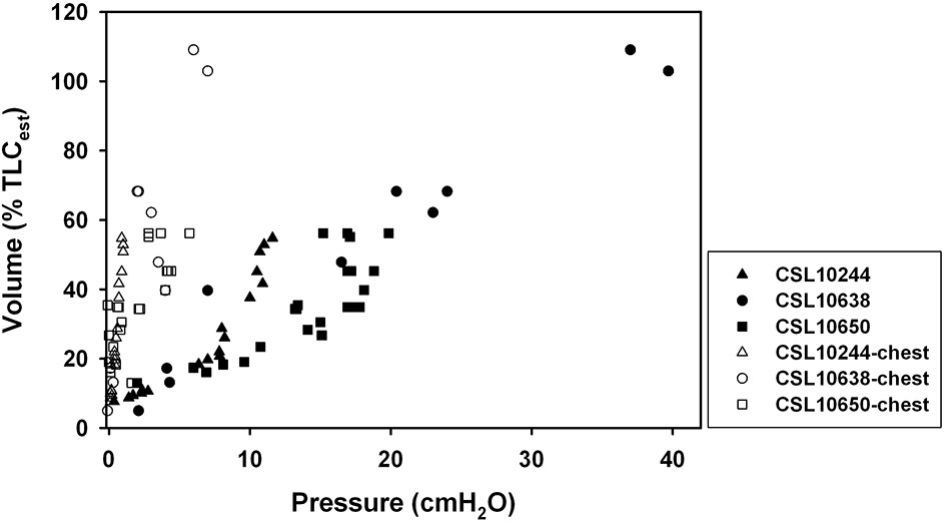
**Relationship between transpulmonary (airway pressure minus esophageal pressure) or chest wall pressure (esophageal pressure minus ambient pressure) and inspired volume expressed as a percent of estimated total lung capacity (Fahlman et al., 2011) for 3 individual California sea lions (*Zalophus californianus*)**.

**Figure 2:**
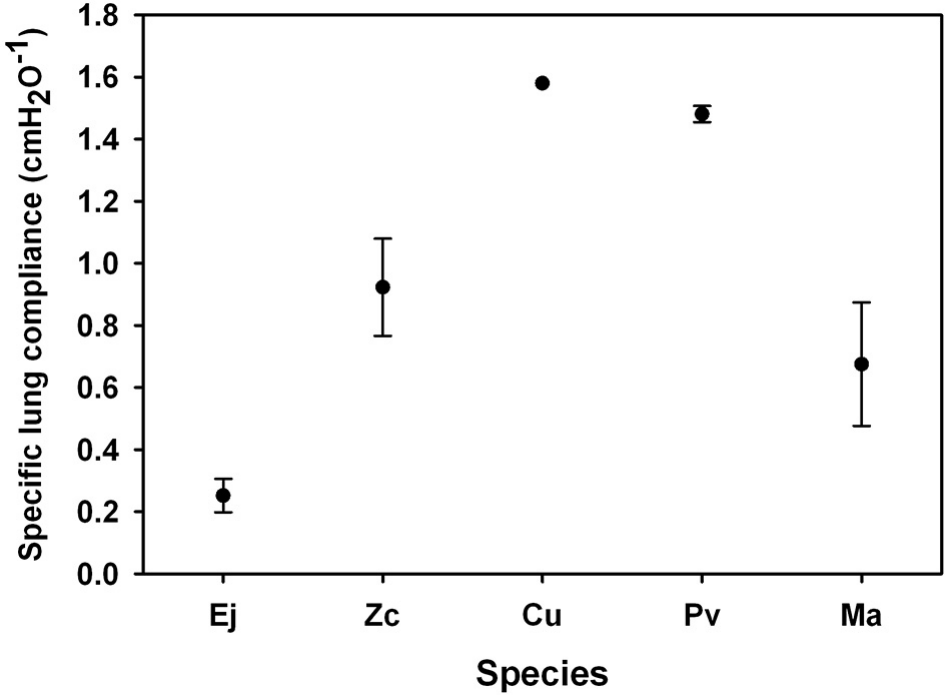
**Average specific lung compliance [the lung compliance divided by the estimated residual volume (RV), where RV was assumed equal to the Minimum Air Volume which was an average 7% of total lung capacity in excised lungs (TLC = 0.135 × Mb^∧^0.92, Kooyman, 1973; Fahlman et al., 2011)] for captive Steller sea lions (Ej = *Eumetopias jubatus*), California sea lion (Zc = *Zalophus californianus*), Northern fur seal (Cu = *Callorhinus ursinus*), harbor seal (Pv = *Phoca vitulina*) and elephant seal (Ma = *Mirounga angustirostris*)**.

**Figure 3:**
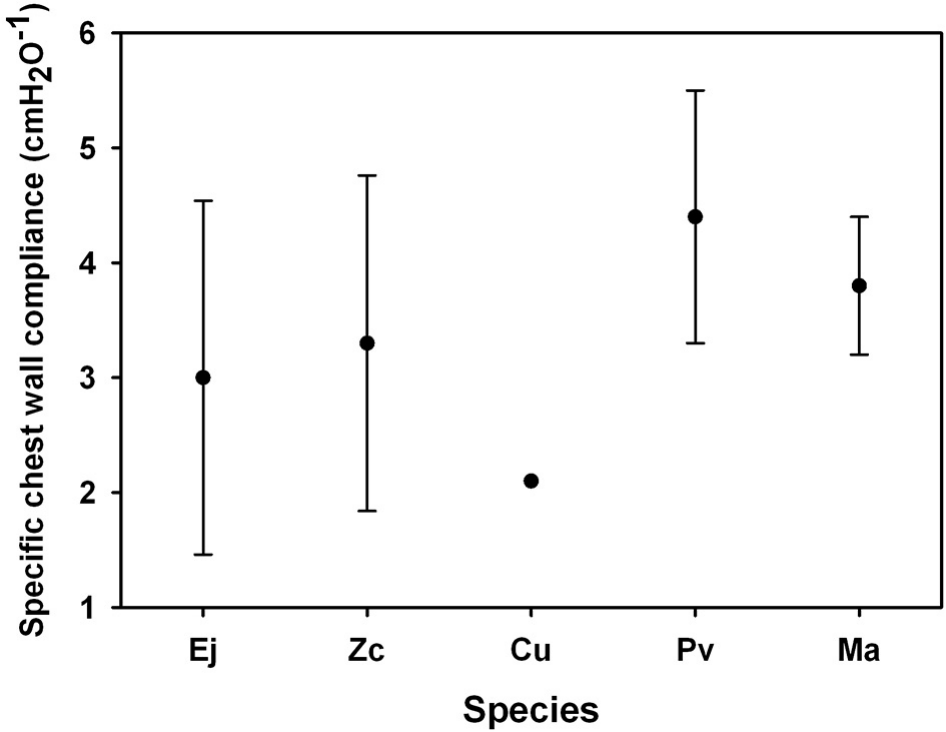
**Average specific chest compliance [the lung compliance divided by the estimated residual volume (RV), where RV was assumed equal to the Minimum Air Volume which was an average 7% of total lung capacity in excised lungs (Fahlman et al., 2011)] for captive Steller sea lions (Ej, *Eumetopias jubatus*), California sea lion (Zc, *Zalophus californianus*), Northern fur seal (Cu, *Callorhinus ursinus*), harbor seal (Pv, *Phoca vitulina*) and elephant seal (Ma, *Mirounga angustirostris*)**.

Morphometrics and static P-V data were collected from 3 otariid and 2 phocid species (Table 1). In all animals, the chest wall compliance (C_CW_) was greater than the lung compliance (C_L_, Figure 1). The sC_L_ was not different in otariids (*n* = 11, 0.617 ± 0.498 cmH_2_O^−1^) as compared to phocids (*n* = 4, 1.078 ± 0.493 cmH_2_O^−1^, One-Way ANOVA, *F* = 0.790, *P* = 0.393, Figure 2). There was a significant correlation between animal size (body mass) and specific lung compliance (sC_L_ = −0.0051 *body mass* + 1.25, *r*^2^ = 0.57, *P* < 0.05), but this relationship was due to the significantly lower specific lung compliance in captive Steller sea lions (0.252 ± 0.131 cmH_2_O^−1^) as compared with wild animals (1.065 ± 0.414 cmH_2_O^−1^, One-Way ANOVA, *F* = 21.2, *P* = 0.0005). There was great variability in the specific chest compliance (sC_CW_) within and among species (3.40 ± 2.50 cmH_2_O, Figure 3) and there were no significant difference among species (One-Way ANOVA *P* > 0.1).

### Whole animal-dead

**Figure 4:**
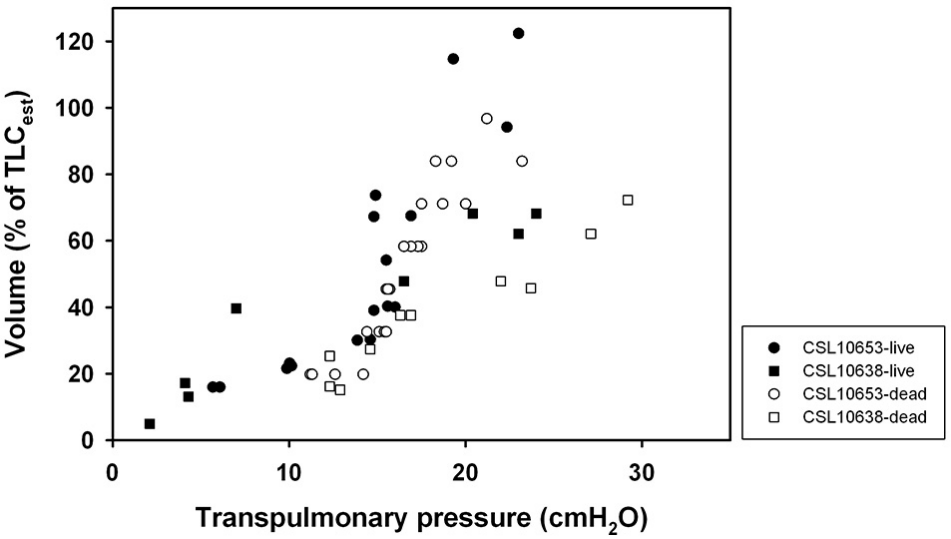
**Relationship between transpulmonary (airway pressure minus esophageal pressure) and inspired volume expressed as a percent of estimated total lung capacity (Fahlman et al., 2011) for alive or deceased California sea lions (*Zalophus californianus*)**.

Lung compliance was estimated in 3 intact California sea lions (CSL10320, CSL10638, CSL10653) post mortem. In two of these, lung compliance data had also been measured before euthanasia and there was good agreement between the measured lung compliances in the live and dead animals (Figure 4, CSL10638 and CSL10653).

### Excised lungs

**Figure 5:**
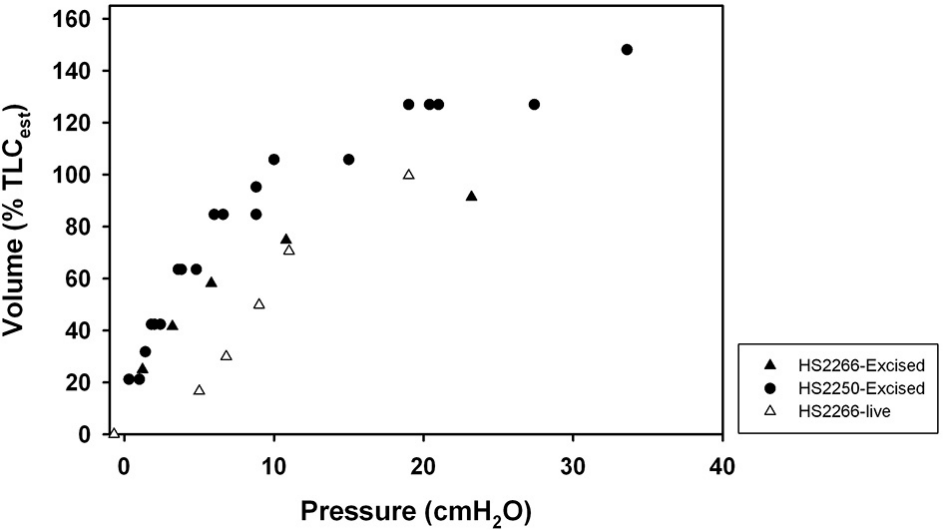
**Relationship between transpulmonary (airway pressure minus esophageal pressure) and inspired volume expressed as a percent of estimated total lung capacity (TLC = 0.135 × Mb^∧^0.92, Kooyman, 1973; Fahlman et al., 2011) for an excised lung and live harbor seal (*Phoca vitulina*). Triangle-HS2266; Circle-HS2250**.

The P-V relationship from excised lungs from 2 harbor seals (HS2250 and HS2266) and 4 California sea lions (CSL10301, CSL10325, CSL10328, and CSL10320, Table 1) were examined. The C_L_ was lower in lungs from smaller animals, but the sC_L_ was similar in all but one animal (CSL10301), where it was twice as high. The P-V relationships of the excised lungs from two harbor seals (HS2266 and HS2250) of similar size were similar (Figure 4). The curve from the live HS2266 was slightly shifted to the right (Figure 5), but had similar slope (Table 1).

## Discussion

Our results suggest that pinnipeds (seals and sea lions) have a highly compliant chest wall that recoils inward to approach the residual volume (RV, Figures 1, 4, 5). Thus, FRC and RV are equivalent in pinnipeds (Fahlman et al., 2011), which supports Scholander's hypothesis that the structural properties of the respiratory system allow the alveoli to compress to the limit of collapse without the risk of lung squeeze (Scholander, 1940; Kooyman, 1973; Kooyman and Sinnett, 1979; Leith, 1989; Fahlman et al., 2011). In addition, we found a significant difference in sC_L_ in pinnipeds from the wild as compared with animals raised in an aquatic facility. These differences suggest that lung conditioning, possibly through repeated diving, may alter respiratory compliance. Alternatively, this incidental finding could indicate decreased lung compliance in older animals.

In 1940, Per Scholander published his seminal work on the physiological adaptations in marine mammals that concluded extended apnea allowed marine mammals to attain great depths. Lung collapse (or more appropriately alveolar collapse) was one trait that would limit gas exchange and uptake of N_2_, and thereby minimize the risk of DCS, or the bends. Scholander suggested that the flexible chest and rigid conductive airways in marine mammals would allow the alveoli to collapse at shallow depths, thereby preventing gas exchange (Scholander, 1940; Leith, 1989). While anatomical descriptions have verified reinforced airways (Scholander, 1940), recent studies have suggested that there may be considerable variability in both anatomy and structural properties in marine mammal tracheas (see references in Tarasoff and Kooyman, 1973).

Our data supplement the few published experimental data (Cozzi et al., 2005; Bagnoli et al., 2011; Moore et al., 2014b) that provide evidence that pinnipeds have compliant chests and lungs that allow compression of the thorax to very low volumes. Respiratory system compliance (C_rs_) is a measure of the lung and chest compliances combined (Leith, 1976), and can be estimated by adding the reciprocal of the individual compliances (1/C_rs_= 1/C_L_+ 1/C_CW_). Consequently, the compliance of the combined system is lower than the individual compliances. For a very compliant structure, the reciprocal approaches 0 and its contribution to C_rs_ decreases. For pinnipeds, with a very high C_CW_ (Figure 1), C_rs_ depends almost entirely on C_L_. Thus, our data support Scholander's hypothesis in pinnipeds and indicate that data on excised lungs should be sufficient to closely estimate C_rs_. Thus, the P-V relationship from excised lungs (Mead, 1961) and conducting airways (Kooyman and Sinnett, 1979; Piscitelli et al., 2010; Fahlman et al., 2011) provide important data that allows prediction of the distribution of air between the alveoli and the airways during diving (Moore et al., 2014b). The lungs and airways, on the other hand, are less compliant, and they will therefore dictate the alveolar collapse depth for a given diving lung volume (Bostrom et al., 2008).

Our study presents data from animals that were anesthetized for various medical procedures in a rehabilitation or aquarium facility. Thus, our sample size and ability to control confounding variables were limited. For example, the available animals were of varying size, gender, and age, which make statistical comparisons limited. In addition, the static P-V relationships were estimated from manual inflations either from a 3 L anesthesia bag or from a ventilator. For animals that were not scheduled for euthanasia, these inflations were conservative to prevent potential alveolar over-inflation and barotrauma. Therefore, the transpulmonary pressures did not always reach 30 cmH_2_O, the pressure often considered to represent total lung capacity (Bostrom et al., 2008). As the P-V relationship is sigmoidal, data limited to the lower portions of the curve may therefore not accurately reflect the compliance at the steep portion of the curve at volumes, which is 50% of TLC. We therefore limited the analysis to measurements where *P*_tp_ > 15 cmH_2_O (Figure 1). While limited in sample size these data provide important information about the respiratory mechanics in pinnipeds and give some valuable insights.

Scholander (Loring et al., 2007) used Boyle's law to estimate the alveolar collapse depth based on the relative size of the upper (trachea and bronchi) and lower airways and alveolar spaces, assuming that the former was incompressible. In 2008, Bostrom et al. (Scholander, 1940) accounted for variation in compliance of the upper and lower airways, and concluded that the alveolar collapse depth, and cessation of gas exchange, may be much deeper if the upper airways are compressed. Empirical evidence for tracheal compression exist in Weddell and elephant seals (Bostrom et al., 2008), and the structural properties of the upper airways appear to extremely varied both within and between marine mammal groups (Kooyman et al., 1970). In addition, studies on post-mortem marine mammals suggest that alveolar collapse depth in marine mammals is probably deeper than formerly estimated (Moore et al., 2014b). As the previous results in post-mortem specimens may have been affected by lack of blood flow and tissue decomposition, we wanted to compare our results with measurements in live animals.

Our results indicate that the chest provides little resistance for compression and that C_rs_ is approximately equal to C_L_. Based on our previous publications (Bostrom et al., 2008; Fahlman et al., 2011; Moore et al., 2011), the new data and the empirical arterial and venous P_O_2__ measurements in sea lions (Bostrom et al., 2008; Fahlman et al., 2009, 2011; Moore et al., 2011, 2014b) suggest that complete alveolar collapse and cessation of gas exchange occur much deeper in most cases than formerly suggested.

It should be kept in mind that physiological adjustments associated with diving (e.g., dive response, blood flow distribution, and thoracic and tracheal blood pooling) (McDonald and Ponganis, 2012, 2013) may also alter the functional compliance. In a diving marine mammal, negative trans-thoracic pressures may cause thoracic blood pooling, similar to that suggested in humans (Leith, 1989). This would help reduce the lung volume and the functional compliance. In other words, thoracic blood pooling would help prevent lung squeeze and its effect on alveolar collapse cannot be assessed in anesthetized animals on land. However, the high sC_CW_ and low MAV prevent extreme negative intra-thoracic pressures from developing and therefore thoracic blood pooling is probably minimal. Several species of marine mammals have a thoracic venous rete that may fill during diving and help compress the lower airways to prevent squeeze. In the striped dolphin (*Stenella coeruleoalba*), blood engorgement of the tracheal lumen may help to alter the internal volume, alter the compliance and help prevent lung squeeze (Craig, 1968; Leith, 1989). Thus, a range of physiological traits may exist in marine mammals that may help prevent respiratory trauma during diving.

### Compliance

The difference in specific compliance between captive and wild raised pinnipeds warrants consideration (Figure 5). Accounting for variation in size showed no significant differences in sC_L_ or sC_CW_ between phocids and otariids. However, sC_L_ but not sC_CW_ differed significantly between the wild animals vs. the Steller sea lions held in aquatic facility (Figure 3), possibly indicating that conditioning may affect lung mechanics. The repeated pressure strain on the chest and lungs during breath-hold diving could help increase the compliance. Consequently, lung conditioning may help alter the mechanical properties of the respiratory system and importantly increase diving fitness. Thus, a pup's ability to dive deep may develop with time as juvenile animals enter the sea and begin diving, thereby increasing the compliance of the lung. This may be similar to the low O_2_ stores in juvenile animals that limits dive duration and that increase with age as the O_2_ stores increase (Baker and Donohue, 2000; Richmond et al., 2006; De Miranda et al., 2012; Moore et al., 2014a).

The Steller sea lions raised at the Aquarium were caught as pups, but were all adults at the time our experiments were conducted with limited diving exposure throughout their life. The wild pinnipeds, on the other hand, were of varying ages. Thus, the variation in specific lung compliance could be due to the differences in age. In humans, physiological aging is known to increase lung compliance and decrease the flexibility of the chest (Richmond et al., 2006). However, the results for lung compliance were opposite to that expected for the older Steller sea lions. These results may indicate that aging may have different effects in marine mammals, and that the respiratory physiology of Steller sea lions is different compared with other pinnipeds, or that life history may help condition the lung. We propose that pinniped pups have moderately compliant chest and lungs, and that conditioning through repeated diving helps increase the compliance and deep diving ability. Increased chest compliance would help reduce potential pulmonary squeeze (Janssens et al., 1999; Janssens, 2005), and together with high lung compliance will alter the pulmonary shunt that develops as the pressure increases during descent (Craig, 1968). Thus, lung conditioning may be vital to altering the diving ability of young animals. Captive animals, on the other hand, have limited ability or incentive for extensive diving, and conditioning may therefore be limited and may explain the lower lung compliance. However, our data set is limited and future work will help test this hypothesis in both humans and marine mammals.

In summary, our data provide experimental evidence supporting Scholander's hypothesis that the chest of pinnipeds is highly compliant and recoils inward toward RV. Thus, the compliance of the upper and lower airways determines the pressure related shunt that develops during diving (Kooyman and Sinnett, 1982; Bostrom et al., 2008; Fahlman et al., 2009), and compliance estimates can be used to determine the alveolar collapse depth (Bostrom et al., 2008). In addition, we found wild animals had higher specific compliance than those raised in an aquatic facility, and suggest that repeated diving may help condition the lung and increase the lung compliance, possibly similar to that found in humans (Bostrom et al., 2008; Fahlman et al., 2009, 2011; Moore et al., 2014b).

### Conflict of interest statement

The authors declare that the research was conducted in the absence of any commercial or financial relationships that could be construed as a potential conflict of interest.
